# Identification of MicroRNAs That Respond to Soybean Cyst Nematode Infection in Early Stages in Resistant and Susceptible Soybean Cultivars

**DOI:** 10.3390/ijms20225634

**Published:** 2019-11-11

**Authors:** Piao Lei, Bing Han, Yuanyuan Wang, Xiaofeng Zhu, Yuanhu Xuan, Xiaoyu Liu, Haiyan Fan, Lijie Chen, Yuxi Duan

**Affiliations:** 1 Nematology Institute of Northern China, Shenyang Agricultural University, Shenyang 110866, China; piaolei9411@163.com (P.L.); hanbing2269@163.com (B.H.); wyuanyuan1225@163.com (Y.W.); syxf2000@163.com (X.Z.); xuanyuanhu007@hotmail.com (Y.X.); fanhaiyan6860@163.com (H.F.); chenlijie0210@163.com (L.C.); 2 College of Plant Protection, Shenyang Agricultural University, Shenyang 110866, China; 3 College of Biological Science and Technology, Shenyang Agricultural University, Shenyang 110866, China; 4 College of Sciences, Shenyang Agricultural University, Shenyang 110866, China

**Keywords:** soybean, soybean cyst nematodes, miRNAs, high-throughput sequencing, early stage

## Abstract

Soybean cyst nematode (SCN) causes heavy losses to soybean yield. In order to investigate the roles of soybean miRNAs during the early stages of infection (1 and 5 dpi), 24 small RNA libraries were constructed from SCN resistant cultivar Huipizhi (HPZ) and the susceptible Williams 82 (W82) cultivar for high-throughput sequencing. By sequencing the small RNA libraries, a total of 634 known miRNAs were identified, and 252 novel miRNAs were predicted. Altogether, 14 known miRNAs belonging to 13 families, and 26 novel miRNAs were differentially expressed and may respond to SCN infection in HPZ and W82. Similar expression results were also confirmed by qRT-PCR. Further analysis of the biological processes that these potential target genes of differentially expressed miRNAs regulate found that they may be strongly related to plant–pathogen interactions. Overall, soybean miRNAs experience profound changes in early stages of SCN infection in both HPZ and W82. The findings of this study can provide insight into miRNAome changes in both HPZ and W82 at the early stages of infection, and may provide a stepping stone for future SCN management.

## 1. Introduction

Soybean [*Glycine max* (L.) Merr.] is rich in oils and proteins, which are in high demand all over the world. Soybean yield is negatively impacted by several soybean pathogens, such as soybean mosaic virus, *Pseudomonas syringae*, *Phytophthora sojae*, *Phakopsora pachyrhizi*, and *Heterodera glycines* [1]. Among these, *Heterodera glycines*, also known as soybean cyst nematode (SCN), causes heavy losses in soybean production of over 120 million USD in China [2] and 1.2 billion USD in the United States [3], making it the most damaging soybean pest worldwide. Therefore, how to restrict the soybean yield losses caused by SCN is a worldwide problem wherever soybean is grown.

To date, SCN is primarily managed by the use of resistant cultivars. In North America, the Peking and PI88788 cultivars have been the predominant resistant sources [4]. Although soybean cultivars conferring resistance to SCN can limit production losses, they can also cause virulence shifts in SCN populations, resulting in SCN with the ability to reproduce on resistant cultivars [5]. Thus, understanding the mechanisms behind soybean–SCN interactions is key in preventing soybean damage from SCN. Although great advances have been made in terms of resistant genes in the form of the serine hydroxymethyltransferase gene (*SHMT*) and quantitative trait loci (QTL) for SCN resistance, such as Resistance to *Heterodera glycines 1* (*Rhg1*) and *Rhg4* [4,6], little is known about the role of soybean microRNA in the soybean–SCN interaction

MicroRNAs are typically endogenous small non-coding RNAs of 20 to 24 nucleotides (nt) in length, which play a crucial role in negatively regulating post-transcriptional gene expression levels [7,8,9]. These small yet impactful plant miRNAs play an important role in orchestrating various biological processes, such as development [10], responses to environmental stress [11], transduction of phytohormone signals [12], and defenses against plant pathogens [13].

In plants, miRNAs originate from *MIRNA* loci, and their biogenesis is an intricate process. Briefly, a *MIRNA* gene is transcripted into single-strand RNAs by RNA polymerase II (pol II) to form a double-strand RNA hairpin structure, known as pri-miRNA. Then, the pri-miRNA is sequentially cleaved into an 80 to 200 nt unstable stem-loop intermediate called the miRNA precursor or pre-miRNA by RNase Ⅲ endoribonuclease Dicer-like 1(DCL 1) enzyme. The pre-miRNA is then directly processed to a miRNA/miRNA* duplex by DCL 1 in the nucleus [7,14,15]. The duplex is exported to the cytoplasm by HASTY, the orthologue of exportin 5. In the cytoplasm, as the miRNA* degrades rapidly, the duplex is separated, and the mature miRNA is incorporated into the ARGONAUTE 1 protein (AGO 1) to form an RNA-INDUCED SILENCING COMPLEX (RISC) [14,15,16]. The AGO1-bound miRNA hybridizes with the target messenger RNA (mRNA) via sequence complementarity, causing degradation or translation inhibition of the corresponding target mRNA [9]. Despite the notable number of *MIRNA* loci in plants, only about 1% of all protein coding genes are targeted by given miRNAs [17]. Plants are at risk of attack from numerous pathogens in the natural environment. To survive, they have evolved a corresponding immune system, involving miRNAs, against these pathogen infections. For example, miR393 is involved in the interaction between *Arabidopsis* and bacterial pathogens through pathogen-associated molecular patterns (PAMPs). This miR393 targets the *TIR1*, *AFB2*, and *AFB3*F F-box family genes, resulting in suppressed auxin signaling and limited *Pseudomonas syringae* growth [18]. In addition, upon bacterial infection, *Arabidopsis* miR167 and 160, which target auxin-related genes, are induced [19]. In *Brassica,* bra-miR1885 responds to *Turnip mosaic virus* (TMV) infection and targets a toll/interleukin-1, nucleotide-binding site leucine-rich repeat (TIR-NB-LRR) disease resistant gene [20]. miR159 and miR166 have been reported as key components in repressing *Verticillium dahlia* damage in cotton [21]. Furthermore, *Arabidopsis miR159abc* mutants were discovered to have lower susceptibility to *Meloidogyne incognita* [22]. Besides their roles in the interactions between plants and pathogens, miRNAs also act as regulators of symbiotic plant–microbe interactions. For instance, in the interaction between *Medicago truncatula* and rhizobia, miR169 regulates the expression of *MtHAP2-1*, which is a key transcriptional factor (TF) in nodule development [23].

As miRNAs play an important role in the plant defense system, current research has focused on the response and function of miRNAs in soybean–SCN interactions. The advancement in deep sequencing technologies, and the rapid growth of bioinformatics, has provided opportunities to gain deeper insights into miRNA regulation of soybean–SCN interactions. Li et al. sequenced four small RNA libraries from cultivars that were resistant and susceptible to SCN race 3, and found that 101 miRNAs were SCN responsive and that 20 miRNAs differed in their expression pattern between resistant and susceptible cultivars [24]. Xu et al. used two sister lines that were resistant and susceptible to SCN race 4 to investigate the responses of miRNAs to infection, and showed that 34 miRNA were expressed differentially in the two lines, and that seven miRNAs were differentially expressed after SCN infection [25]. Tian et al. reported that a total of 60 miRNAs belonging to 25 families might be related to cultivar-specific responses to SCN [26].

Despite these studies that have investigated how soybean miRNAs respond to SCN infection and contributed to our understanding of the role of miRNAs in responding to SCN invasion, the responses of miRNAs in the early stages of SCN infection remain unknown, due to the fact that early stages are essential for SCN to successfully reach the vascular tissue of their host root where SCN establish an initial feeding site and absorb enough nutrients to reach their reproductive stage. Therefore, the changes in soybean miRNA responses to SCN in the early stages of infection need to be investigated, and it will be valuable to have a detailed understanding of soybean–SCN interactions at the miRNA level, due to the fact that soybean miRNAs play a key role in soybean disease defenses [27]. Here, we sequenced 24 miRNA libraries from an elite resistant soybean cultivar (Huipizhi) that confers resistance to diverse SCN races (race 1, 3, 4, 5, 7, and 14) [28] and from a susceptible cultivar (Williams 82) at 1 and 5 dpi of SCN infection.

## 2. Results

### 2.1. HPZ Was Highly Resistant to SCN During the Early Stages of Infection/HPZ Represses the Development of SCN and Syncytium

**Figure 1:**
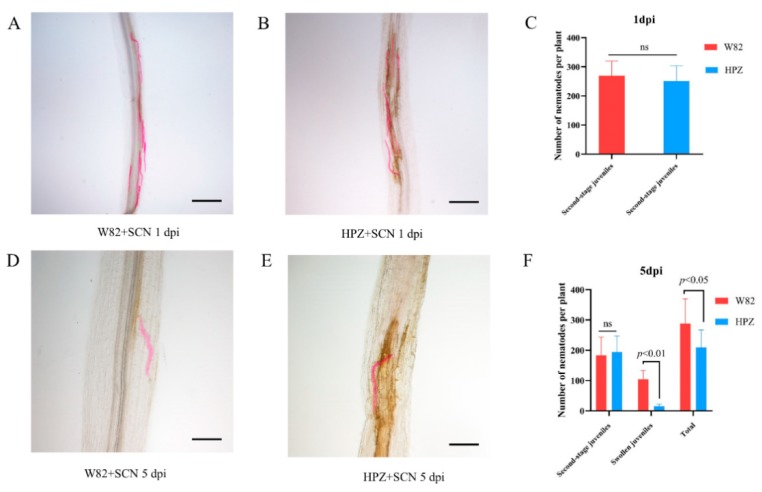
SCN (soybean cyst nematode) juveniles; infection rate and development state assay in HPZ and W82. Each soybean seedling was inoculated with 2000 second-stage SCN juveniles, and the infection rate was assayed at 1 dpi in W82 and HPZ (**A**–**C**), A: second-stage SCN juveniles infected the root of W82, bar = 500 μm, B: second-stage SCN juveniles infected the root of HPZ, bar = 500 μm, C: total number of second-stage SCN juveniles infected the root of W82 and HPZ, values are the mean of the number of SCN, bar, standard error. The development state was assayed at 5 dpi (**D**–**F**), D: SCN juveniles developed into the swollen stage in W82, bar =200 μm, (**E**) the development of SCN juveniles was delayed in HPZ, bar = 200 μm, (**F**) total number of SCN different state juveniles in W82 and HPZ at 5 dpi, values are the mean of the number of SCN, bar, standard error. Pictures of SCN juveniles in the soybean root were taken by an OLYMPUS DP80 light microscope (Olympus, Tokyo, Japan). Nematode infection and development data were checked for normality with the Kolmogorov–Smirnov or Shapiro–Wilk test, then the t-test method was used to analyze these above data with SPSS software. Each soybean cultivar contained 10 replicates, ns: not significant, *p* < 0.05, *p* < 0.01 means a significant difference was found between each comparison.

**Figure 2:**
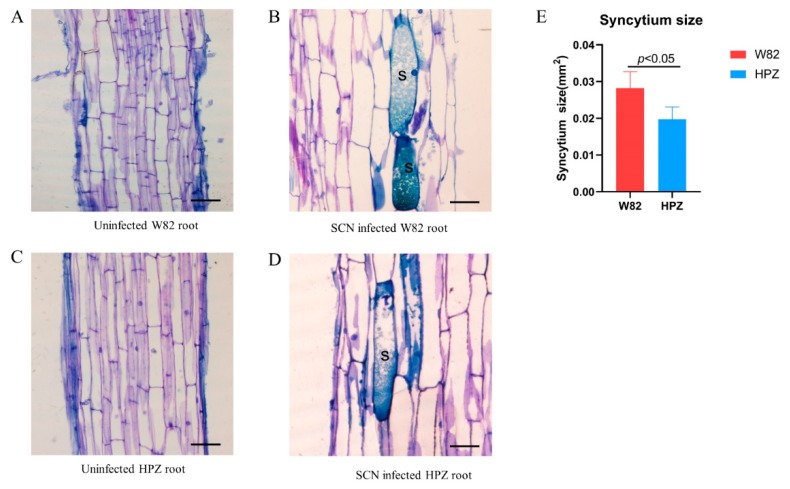
Histological observation of syncytium size in HPZ and W82 at 5 dpi. (**A**) uninfected W82 root, bar = 100 μm, (**B**) SCN-infected W82 root, the syncytium induced by SCN was labeled with “S”, bar = 50 μm, (**C**) uninfected HPZ root, bar = 100 μm, (**D**) SCN-infected HPZ root, the syncytium induced by SCN was labeled with “S”, bar = 50 μm, (**E**) average size of syncytium in W82 and HPZ values are the mean syncytium size, bar, standard error. Pictures of syncytia were taken by an OLYMPUS DP80 light microscope (Olympus, Japan), and the size of the syncytia was measured with Cellsens standard software. Syncytium size data were checked for normality with the Kolmogorov–Smirnov or Shapiro–Wilk test, then the t-test method was used to analyze these above data with SPSS software. Size values of the five syncytia were used for t-test, *p* < 0.05 means a significant difference was found between W82 and HPZ.

The HPZ soybean cultivar has a broad resistant spectrum to several SCN races (race 1, 3, 4, 5, 7, and 14) [28]. To investigate the infection and development of SCN (race 3) in the roots of the HPZ and W82 cultivars during the early stages of infection (1 and 5 dpi), we inoculated each HPZ and W82 seedling with 2000 SCN J2s, and the infection rate and development state were recorded at 1 and 5 dpi. There were no significant differences in the total number of SCN juveniles in the roots of HPZ and W82 at 1 dpi (Figure 1A–C). However, nearly 50% of SCN juveniles in W82 had developed to the swollen stage at 5 dpi, compared to only 10 % in HPZ (Figure 1D–F). The fact that development of SCN was delayed in HPZ may be related to the establishment and development of syncytium. Therefore, we also measured the size of syncytium that SCN induced in the roots of HPZ and W82 at 5 dpi. The average syncytium size in W82 was 0.028 mm^2^, which was 1.5 times greater than the HPZ average (0.019 mm^2^) at 5 dpi (Figure 2), and this difference was significant (*p* < 0.05). This result also suggests that resistant soybean cultivars do not impact the penetration of SCN, but that they instead degenerate and restrict the formation and development of syncytia using multiple mechanisms in the early stages of soybean–SCN interactions [29,30,31].

### 2.2. Small RNA Sequenced from the 24 Libraries Constructed from HPZ and W82 Roots

**Figure 3:**
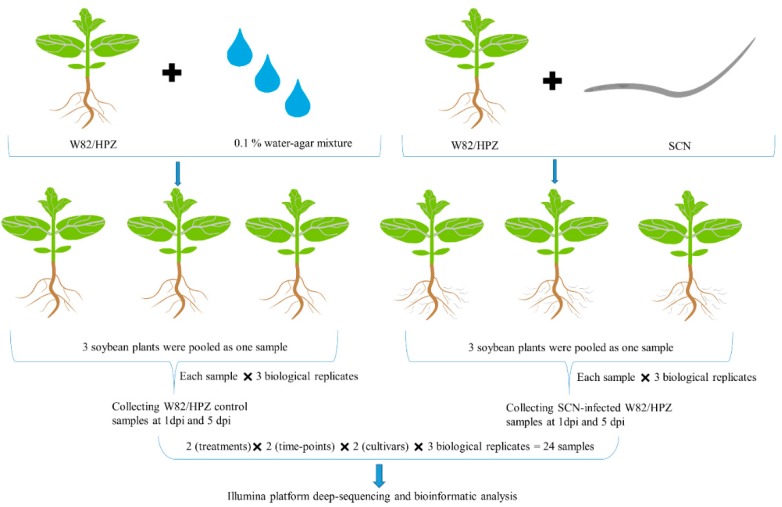
The workflow of the construction of 24 small libraries. In total, 1 mL 0.1% water-agar mixture containing approximately 2000 SCN J2s (second stage juveniles) or water-agar mixture alone as the treatment or control, respectively, were added to the root system of soybean seedlings. Three soybean seedlings were pooled as one sample, each sample contained three biological replicates, and these samples were collected at 1 and 5 dpi, respectively. In total, 24 samples used for small RNA libraries construction were prepared, namely, 24 samples = 2 (treatments) × 2 (time-points) × 2 (cultivars) × 3 (biological replicates).

Despite HPZ conferring strong resistance to SCN in the early stages of SCN parasitism, until now the changes and roles of miRNAs during this important stage remain to be elicited. Toward this goal, we constructed 24 small RNA libraries to sequence using an Illumina novaseq6000 platform, and a workflow diagram of library construction is shown in Figure 3. These 24 libraries provided a total of 1,020,975,683 sRNA raw reads, and the Q30 of all the libraries was ≥94%. After data quality control, a total of 643,468,338 sRNA clean reads were obtained, and each library contained a minimum of 19,622,055 clean reads (Table 1). Then, these clean reads were used for sRNA annotation and no less than 17.07% of the clean reads in each sample were using to detect known and novel miRNAs (Material S1).

### 2.3. Identification of Known and Novel miRNAs

To detect known miRNAs, the remaining unannotated reads were compared with the soybean reference genome (*Glycine max*.Wm82.a2.v1) [32] and miRbase (v22) [33]. In total, 634 known miRNAs were identified and 252 novel miRNAs were predicted across all 24 libraries (Table 2). Several legume-specific known miRNAs were identified in soybean in all libraries, including gma-miR10186a (10186b, 10186d, 10186f), gma-miR10187, and gma-miR10193, which were all weakly expressed in both HPZ and W82 regardless of whether plants were infected with SCN at 1 and 5 dpi. Contrastingly, gma-miR10440, gma-miR1508a (1508b, 1508c), and gma-miR1510a (1510b) had high expression levels in all libraries. Conserved miRNAs, such as gma-miR166, gma-miR171k, gma-miR396b-5p, gma-miR398d, and gma-miR482b, were also highly abundant in all libraries, while gma-miR390b-3p and gma-miR393a (393b) were weakly expressed in all libraries. Novel_miR224, novel_miR128, and novel_miR76 were highly abundant in all libraries (Material S2). Perhaps these highly expressed miRNAs play important roles in regulating basic biological processes at 1 and 5 dpi in both HPZ and W82, and miRNAs with low expression levels may have a contrary position in this period.

### 2.4. Analysis of Differences in miRNA Expression Between HPZ and W82

To identify miRNAs responsive to SCN infection in both HPZ and W82 at the early stages of infection (1 and 5 dpi), miRNA differential expression (DE) profiling was completed between control groups (W1C, W5C, H1C, H5C) and SCN-treated groups (W1N, W5N, H1N, H5N) at different time points (1 and 5 dpi) in both HPZ and W82.

**Figure 4:**
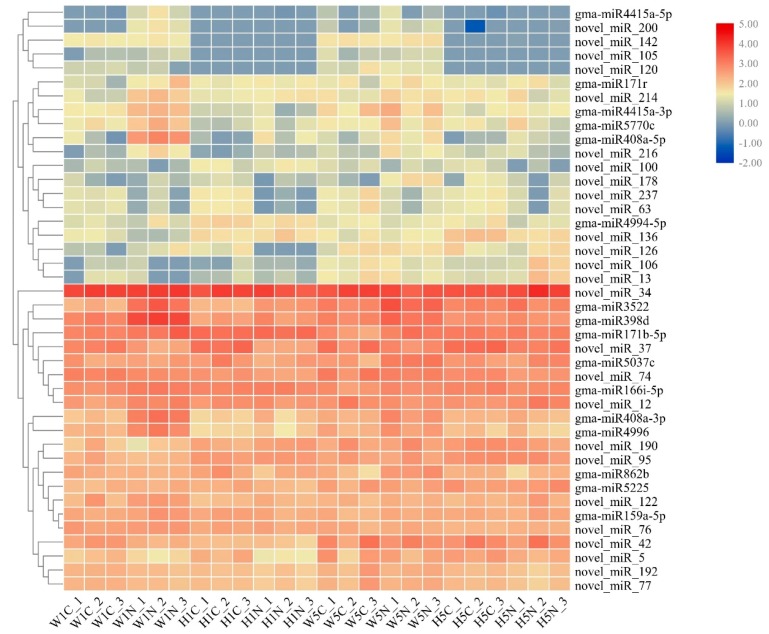
Hierarchical clustering analysis of all the differentially expressed miRNAs. The differentially expressed miRNAs were analyzed by hierarchical clustering base on log10 (TPM + 10^−6^) values of miRNAs, and the miRNAs with the same or similar expression pattern were clustered. The columns represent different samples and rows represent different miRNAs, clustered with values, with red representing high miRNA expression and blue representing low miRNA expression.

A total of 14 known miRNAs belonging to 13 families, and 26 novel miRNAs were differentially expressed in the 24 libraries (Material S3). Eleven miRNAs were differentially expressed between the susceptible cultivar W82 control library and the SCN-treated library at 1 dpi, while only three miRNAs (gma-miR3522, gma-miR408a-3p, and novel_miR_178) were upregulated at 5 dpi. Between the HPZ control library and the HPZ SCN-treated library, seven miRNAs were up- or downregulated at 1 dpi, and only three novel miRNAs (novel_miR_106, novel_miR_13, and novel_miR_34) were upregulated. We also conducted DE miRNAs analysis of the same cultivar infected by SCN at different time points in order to identify the soybean miRNAs that changed with SCN development. Eight miRNAs were downregulated in W5N compared to W1N, including conserved miRNAs, such as gma-miR408a, gam-miR171, gma-miR159, and gma-miR398d, while three miRNAs were upregulated, including gma-miR5037c and novel_miR_190. Six miRNAs were upregulated in H5N compared to H1N, including gma-miR5225, while three miRNAs were downregulated, such as miR171b-5p. To find the miRNAs that were differentially expressed between the resistant cultivar HPZ and the susceptible cultivar W82, we compared the SCN-infected libraries of the two cultivars. We found that three miRNAs were upregulated (novel_miR95, novel_miR190, and novel_miR136), and seven miRNAs were downregulated, including gma-miR408a, novel_miR120, and novel_miR214, in W1N compared to H1N. Nine miRNAs were downregulated and no miRNAs were upregulated in W5N compared to H5N (Table 3 and Material S3). After DE analysis of miRNAs in all DE sets, a hierarchical clustering analysis was performed on all DE miRNAs using TBtools software [34] based on the value of log10 (TPM1e-6). miRNAs with the same or similar expression behaviors were clustered. As shown in Figure 4, gma-408a seemed to respond strongly to SCN infection in the W82 susceptible cultivar at 1 and 5 dpi, whereas gma-4994-5p responded to SCN infection in the HPZ resistant cultivar at 1 and 5 dpi. We could deduce that gam-398d, gma-159a-5p, and novel_miR_34 responded to SCN infection and were highly expressed (Figure 4).

**Figure 5:**
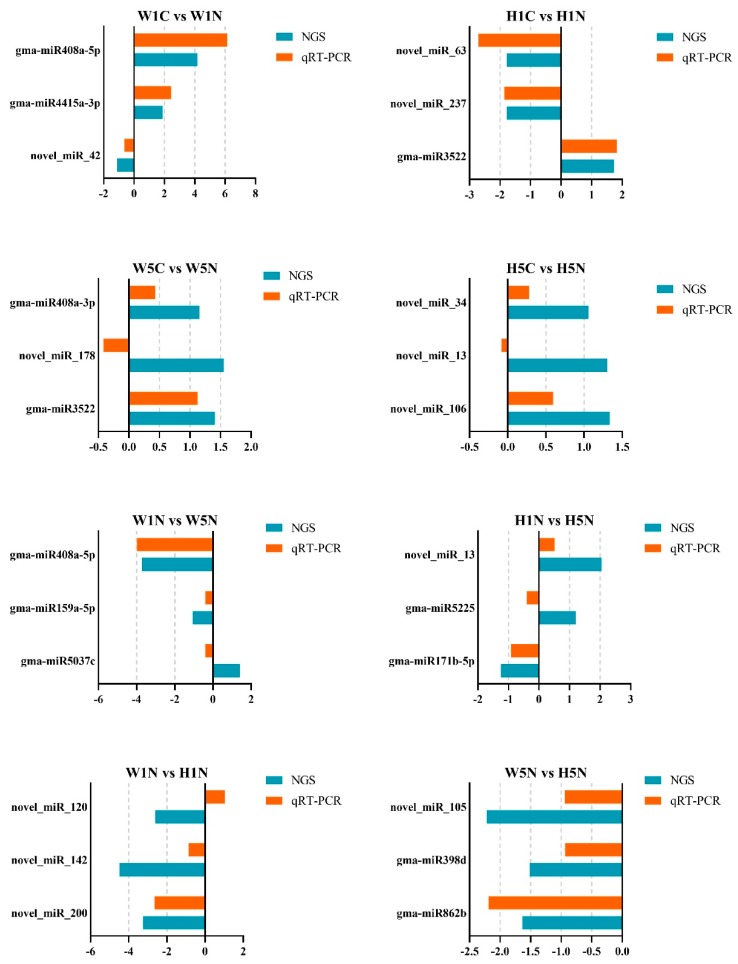
qRT-PCR validates the expression pattern of selected miRNAs in high-throughput sequencing. Three miRNAs in each comparison were randomly selected for qRT-PCR validation, X axis stands for relative expression of miRNAs, NGS, the expression pattern of miRNAs obtained in high-throughput sequencing, qRT-PCR, and the miRNA expression pattern of miRNAs validated by qRT-PCR.

To validate the sequencing data and our DE analysis of miRNAs, we conducted qRT-PCR using the same 24 RNA samples. In each DE set, three differentially expressed miRNAs found in silico were randomly selected for qRT-PCR experiments. The results of the qRT-PCR showed that the expressions of most of the miRNAs (19 of 24 selected miRNAs) agreed with the sequencing result. For instance, gam-miR408a-5p expression was 4.1 times higher in W1N compared to W1C according to the NGS result (Material S4), and qRT-PCR found that its expression was 6.1 times higher (Figure 5). The same consistent expression patterns could be found for other comparisons, like gma-miR3522 expression between H1C and H1N, and gma-miR398d expression between W5N and H5N (Material S4 and Figure 5).

### 2.5. Target Prediction and Function Annotation

miRNAs exert their regulatory roles by interaction with transcripts of their target genes. In this way, miRNAs regulate diverse biological processes within the plant. To understand the biological changes that miRNAs regulate in soybean in response to SCN infection in both HPZ and W82, we used TargetFinder (v1.6) to predict the potential target genes of the identified DE miRNAs. A total of 8487 potential target genes were predicted (Material S4), before they were annotated by the Blast to the NR, Swiss-Prot, GO, KEGG, and Pfam databases. We noticed that the target genes of DE miRNAs were strongly linked to plant defense processes in both W82 and HPZ. For example, gma-miR408a regulated several genes that function as xylanase inhibitors, protein tyrosine kinases, and ubiquitin-conjugating enzymes, and gma-miR159a regulated genes with GAMYB domains or NB-ARC domains, which program cell death and pathogen responses. In addition, gma-miR398d regulated genes with copper/zinc superoxide dismutase (SODC) functions, gma-miR862b regulated genes encoding serine hydroxymethyltransferase, which is a key gene in *Rhg* loci, and gma-miR4415 was predicted to regulate several multicopper oxidase genes. Several novel miRNAs were predicted to regulate signal transduction- and phytohormone-related genes. For instance, novel_miR_106 regulated genes related to K+ potassium transporters, novel_miR_13 regulated genes with leucine-rich repeat domains, novel_miR_34 regulated genes encoding auxin response factors, and novel_miR_178 regulated genes with AP2 ethylene-responsive transcription factor domains (Material S4). These miRNAs may play essential roles in the interactions between SCN and both the HPZ resistant cultivar and the susceptible W82 cultivar (Table 4 and Material S4).

**Figure 6:**
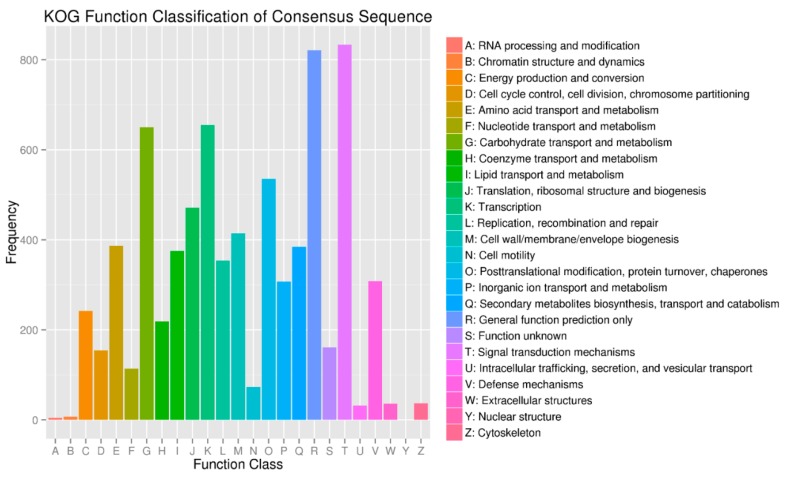
KOG function classification of target genes of differentially expressed miRNAs. The Y axis stands for the frequency of target genes while the X axis stands for the function classification of target genes.

**Figure 7:**
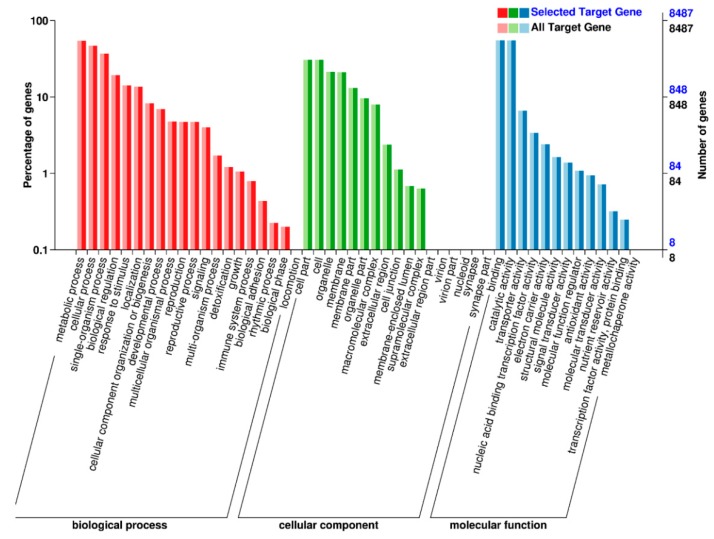
GO function classification of target genes of differentially expressed miRNAs. The left Y axis stands for the percentage of target genes, and the right Y axis stands for the number of target genes and the X axis stands for the function classification in biological process, cellular components, and molecular function.

**Figure 8:**
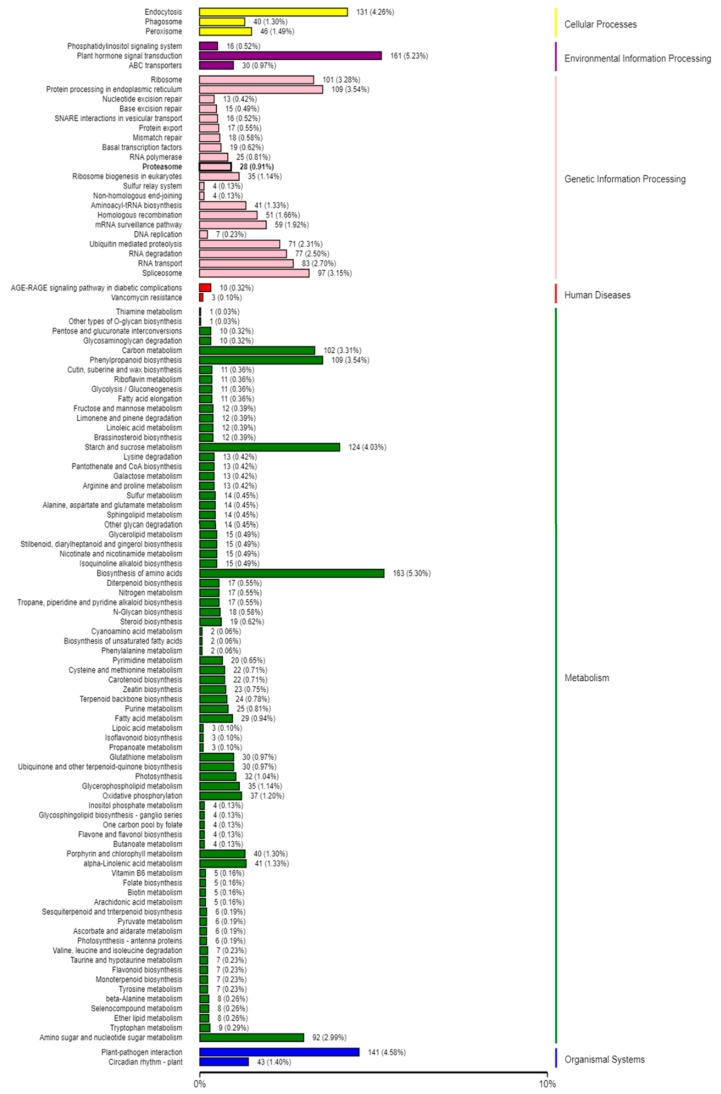
KEGG pathway classification of target genes of differentially expressed miRNAs. The left Y axis is the name of the KEGG metabolic pathway, the right Y axis is the biological process that these genes participated, and the X axis is the number and the percentage of target genes.

In order to gain insight into the biological processes that are influenced by the SCN infection in both HPZ and W82, we conducted KOG, GO, and KEGG pathway classification for the biological processes influenced by these potential target genes using a BMK cloud analysis platform. As expected, we found that signal transduction mechanisms, carbohydrate transport, metabolism, and defense mechanisms occurred very frequently in KOG classification (Figure 6). In GO classification, immune system processes and responses to stimulus detoxification were found at high percentages in biological processes. Antioxidant activity, nutrient reservoir activity, and signal transducer activity occurred frequently in the molecular function (Figure 7). In KEGG pathway classification, 4.58% of genes were involved in plant–pathogen interaction in the organism systems, 5.30% of genes participated in amino acid biosynthesis, 4.03% were involved in starch and sucrose metabolism, and 3.54% played roles in phenylpropanoid biosynthesis (Figure 8).

## 3. Discussion

Plant miRNAs play an active role in regulating diverse biological processes, including responses to biotic and abiotic stresses, and many recent studies have provided evidences of this. Jiang et al. found that miR482b negatively impacted tomato resistance to *Phytophthora infestans*, with plants overexpressing miR482b displaying more serious disease symptoms [35]. Dai and coworkers proved that OsmiR396 negatively impacted rice resistance to brown planthoppers [36], and Ding et al. found that overexpression of miR160 could increase the sensitivity of cotton to high temperature stress [37]. Plant miRNAs also strongly respond to parasitism by plant parasitic nematodes. In *Arabidopsis*, Hewezi and coworkers studied the relationship between *Arabidopsis* miRNAs and parasitism by the beet cyst nematode (*Heterodera schachtii*) by sequencing the miRNA libraries constructed from the syncytia formed in *Arabidopsis* roots using LCM (laser capture microdissection) technology. Their results identified the miRNAs that were differentially expressed compared to control roots, and syncytia were predicted to regulate genes coding for transcription factors that regulate hormonal responses, cellular specialization, or cell expansion [38]. Several miRNAome sequencing experiments have since been conducted to investigate how plant miRNAs change when the plant is infected by parasitic nematodes, including studies on *Arabidopsis* infected with the root knot nematode *M. javanica* [39], *Arabidopsis* infected with *M. incognita* [22], tomato infected with the potato cyst nematode *Globodera rostochiensis* [40], and tomato infected with *M. incognita* [41]. Soybean cyst nematodes are one of the most devastating pathogens of soybean, casing heavy losses to soybean production worldwide [2,3]. To understand the function of miRNAs displayed during soybean SCN infection, three miRNAome changes experiments were conducted by SCN researchers. Li et al. investigated the miRNAs involved in infection by SCN race 3 using a resistant cultivar (HB) and a susceptible cultivar (L10). They found that 101 miRNAs were SCN responsive and that 20 miRNAs differed in their expression patterns between resistant and susceptible cultivars. Xu and coworkers sequenced miRNAome changes between soybean roots infected with SCN race 4 and control roots from resistant and susceptible cultivars. Their results showed that seven miRNAs were differentially expressed after SCN infection [25]. These previous studies provide us with new insight into how miRNAs respond to SCN infection; however, due to these studies lacking biological replicates, there may be some bias caused by different expression analysis methods. Recently, Tian et al. used two soybean cultivars (SCN susceptible KS4607 and SCN HG Type 7 resistant KS4313N) that were grown under SCN-infested and non-infested soil at two different time points (SCN feeding establishment and egg production). In their study, each treatment contained three replicates, and they identified 60 miRNAs that were specifically related to different resistant cultivars when infected by SCN [26]. To understand the miRNA changes at the early stages of infection, in our present study, we constructed 24 small libraries from W82 control plants, W82 plants infected by SCN, HPZ control plants, and HPZ plants infected by SCN at the early stages of SCN infection (1 and 5 dpi; namely W1C, W1N, H1C, H1N, W5C, W5N, H5C, and H5N, the workflow is shown in Figure 3). Each treatment consisted of three replicates in order to achieve high confidence in our high-throughput sequencing results because of the different expressions of miRNAs among each replicate, as shown in Supplementary Materials S2 and S3. Using the Illumina Novaseq6000, we obtained a total of 643,468,338 sRNA clean reads after removing the low-quality reads and adapters, which is greater than the 13,835,079 clean reads obtained in Xu et al. [25], the 44,515,792 reads reported in Li et al. [24], and the 58,628,603 reads in Tian et al. [26].

In this study, owing to the large number of reads obtained by Illumina Novaseq6000, a total of 634 known miRNAs were identified and 252 novel miRNAs were predicted. The most important aspect of high-throughput sequencing of miRNA changes is different expression analysis. We normalized the expression level of each miRNA using TPM, and used DEseq2 software to find miRNAs that were differentially expressed by setting the |log2(FC)| ≥ 1.00; FDR (False Discovery Rate) ≤ 0.01. We found 14 known miRNAs belonging to 13 families, and 26 novel miRNAs were differentially expressed in these 24 libraries (Supplementary Material S3). Our results differed greatly from those of other studies in terms of the number of DE miRNAs or families of miRNAs. This may be due to the specific soybean cultivars used for sequencing, the time points at which we collected the samples, and the software used for analysis [24,25,26]. In this study, there were more DE miRNAs in the susceptible soybean cultivar and the resistant cultivar at 1 dpi compared to 5 dpi. No miRNAs were downregulated, but only three miRNAs we found to be differentially expressed in comparison between W5C and W5N and between H5C and H5N, and the fold-changes of all DE miRNAs at 5 dpi were less than 1.5 (Table 3 and Material S4). This is an interesting phenomenon in our sequencing results, and our qRT-PCR confirmed the expressions of most of the DE miRNAs between W5C and W5N and between H5C and H5N (Figure 7). The fact that more miRNAs were differentially expressed in both HPZ and W82 at 1 dpi compared to 5 dpi could be explained by extensive tuning of the host’s response to nematode infection [42]. Koter et al. reported a sharp increase in host miRNA synthesis in the first days after nematode infection in tomato [40]. However, studies have shown that more miRNAs are upregulated in the late stages of nematode infection in soybean [26] and in *Arabidopsis* [38]. We considered that these differences may come from the type of plant, specific nematode, and the time points measured in these studies. Therefore, differences between sequencing experiments are a normal phenomenon.

miRNAs exert control by influencing the expressions of target genes. In order to further understand which genes of soybean cultivar HPZ and W82 were regulated by miRNAs during soybean root SCN infection, we used TargetFinder (v1.6) to predict the potential target genes of the identified DE miRNAs. The legume-specific gma-miR3522, which was upregulated in W82 at 1 and 5 dpi and in HPZ at 1 dpi, was predicted to negatively control several genes encoding polyphenol oxidase (Material S4), which enhances the resistance of tomato to *Pseudomonas syringae* [43]. Of the other legume-specific miRNAs, gma-miR5037c was upregulated in W5N compared to W1N, and targets genes encoding GMC oxidoreductase, which is reportedly involved in the biocontrol activities of several plant pathogenic fungi [44]. gma-miR5225 was differentially expressed in the H1N vs. H5N comparison, and targets genes coding for protein tyrosine kinase, which plays essential roles in plant disease resistance, stress responses, and cell death in *Arabidopsis* [44]. Little is known about the function of these legume-specific miRNAs in soybean, and their roles remain to be elucidated, especially in terms of how they impact the interactions between soybean and SCN.

The function of conserved miRNA is relatively well understood, as they are highly conserved in function and structure. In our study, conserved miRNA gma-miR408a was found to respond to SCN infection in W82 at 1 and 5 dpi, and in HPZ at 1 dpi. On the other hand, Tian et al. found that gma-miR408a only responded to SCN infection in a susceptible cultivar [26]. The potential target genes of gma-miR408a encode multicopper oxidase, which contributes to plant resistance to several plant pathogens [45]. Furthermore, gma-miR159a and gma-miR398d were both differentially expressed in the comparison between W1N and W5N. gma-miR159 was reported to play a role in plant resistance to *P. syringae* and regulates the GAMYB transcript factor [46,47], while gma-miR398d targets genes coding for copper/zinc superoxide dismutase (SODC), which participates in plant responses to heat stress and barley powdery mildew fungus [48,49]. Downregulation of gma-miR398 induces the plant to produce superoxide dismutase 1 (CSD1) and superoxide dismutase 2 (CSD2), which improve tolerance to oxidative stress in *Arabidopsis* [50]. In our study, 252 novel miRNAs were predicted, and different expression analysis showed that several novel miRNAs were differentially expressed between W82 and HPZ, indicating that they may play important roles in responding to SCN infection. For example, novel_miR_178 was upregulated in W5N compared to W5C, which targets AP2/ EREBP (APETALA2/ethylene-responsive element binding protein) transcripts. It has been reported that overexpression for AP2/EREBP enhanced resistance to *Phytophthora parasitica* in tobacco [51]. Novel_miR_106 was upregulated in H5N compared to H5C, which targets several genes coding for leucine-rich repeat domains. This domain belongs to typical resistant genes (R gene), which play a positive role in inhibiting the invasion of plant pathogens [52]. The target genes of the DE miRNAs in every comparison mentioned above appear to be potential soybean miRNA candidates that are responsive to SCN infection in both HPZ and W82 in the early stages of infection.

In present study, we conducted KOG, GO, and KEGG pathway classification of these predicted target genes to gain insight into which soybean biological processes were altered by SCN infection. We found that the immune system, stimulus detoxification, plant–pathogen interaction, and phenylpropanoid biosynthesis occupied a high percentage in the KOG, GO, and KEGG pathway classifications. These terms were highly related to plant defense, for example, the phenylpropanoid biosynthesis term was reported to be involved in plant resistance to disease [53]. Further, nutrient reservoir activity, signal transducer activity, biosynthesis of amino acids, and starch and sucrose metabolism also constituted high percentages in these classifications, indicating that the soybean metabolism was dramatically altered in response to SCN invasion. As Siddique and Grundler reviewed, in nematode feeding sites, nutrients like amino acids experience profound changes, and sucrose and starch are increased during nematode development [54]. These metabolic changes to the target genes of the DE miRNAs may increase our understanding of the response of both HPZ and W82 to SCN.

As has been mentioned in this paper, several experiments were conducted to understand host plant miRNAome changes during the plant parasitism by nematodes. These studies focused on the miRNAome changes caused by CN (cyst nematode, *Heterodera* spp.) [24,25,26,40] and RKN (root knot nematode, *Meloidogyne* spp.) [22,38,39,41]. To date, the function of miR159, miR172, and miR319 in the interaction between plants and plant parasitic nematodes are the few miRNA functions that have been deeply elucidated [22,55,56], and many questions remain surrounding the roles of miRNAs in plant parasitism by nematodes. For instance, cross-kingdom RNA silencing probably also occurs during interactions between plants and plant parasitic nematodes [57]. In other plant species, recent studied have indicated that plant miRNAs can silence the transcripts of plant pathogens that are important for virulence [21], and that miRNAs from the plant pathogen also can silence genes of their hosts that are important for immunity [58]. The question remains as to whether soybean miRNAs silence SCN genes that are important for parasitism. Combining high-throughput sequencing technologies and novel technologies like short tandem target mimic (STTM), which has already been applied to investigate the functions of plant endogenous miRNAs in *Arabidopsis*, soybean, and tomato [35,59,60], and CRISPR/Cas9, a powerful tool for creating soybean mutants [61], with the large datasets obtained in miRNAome sequencing studies will allow the concrete roles of specific miRNAs in the interaction between SCN and soybean to be understood. Then, the regulatory roles of miRNAs can be used respond to SCN as a powerful, highly efficient strategy to improve the yield and resistance of soybean to SCN.

## 4. Materials and Methods

### 4.1. Soybean, Nematode Population Culture

*Glycine max*, “Huipizhi” (HPZ) and Williams 82 (W82) were selected as resistant and susceptible cultivars to SCN race 3, respectively. Soybean seeds were surface sterilized with 1% NaClO for 10 min and were washed at least five times with sterilized water. Then, the seeds were geminated in PVC tubes (height × width = 10 × 3 cm) containing equal ratios of sterilized sand and soil in a climatic chamber (light/dark = 16/8, 23–26 °C, 50% relative humidity). Hoagland’s nutrient solution was added to soybean seedlings once every three days. The *Heterodera glycines* (soybean cyst nematode, SCN) race 3 population was propagated on W82 in infested soil in a greenhouse in the Nematode Institute of Northern China (Shenyang, China). After two months, SCN cysts were extracted from the infested soil on a 60-mesh sieve (250 μm), and harvested cysts were then crushed on an 80-mesh sieve (180 μm). Eggs were collected on a 500-mesh sieve (25 μm), and were further purified using 35% (*w*/*v*) sucrose solution. Eggs were sterilized with 0.1% NaClO for 10 min before being rinsed with sterilized water several times to remove any traces of NaClO. The sterilized eggs were transferred to a modified Baermann pan with 3 mM ZnSO_4_ at 25 °C in the dark for 5 days to allow them to hatch, and the freshly hatched pre-parasitic second-stage juveniles (J2s) of SCN were then harvested [62,63].

### 4.2. Nematode Inoculation, Penetration, Development Evaluation, and Histological Observation

Ten days after soybean seedlings geminated, a hole was dug around the soybean seedlings. One milliliter of 0.1% water-agar mixture containing approximately 2000 SCN J2s or water-agar mixture alone as a treatment or control, respectively, were added to the root systems of soybean seedlings, and these seedlings were grown under the conditions described above. Infections were synchronized by washing the infected roots at 24 h post-inoculation.

To analyze the penetration and development of SCN in the roots of HPZ and W82, 10 roots of each cultivar were harvested at 1 and 5 dpi, and were stained using the method reported in Bybd et al. [64]. Then, SCN juveniles were counted using a Nikon SMZ800 stereomicroscope (Nikon, Japan). To observe the histological changes in HPZ and W82 at 1 and 5 dpi, SCN-infected and non-infected roots of HPZ and W82 were cut into 1-cm cubes before being put into Carnoy’s fix solution, dehydrated with gradient ethanol, and embedded in Technovit 7100 (Heraeus Kulzer, Hanau, Germany) according to the manufacturer’s instructions. Embedded roots were sectioned (3 μm) using a Leica CM1860 UV microtome (Leica, Wetzlar, Germany) before being stained by 1% toluidine blue (Sigma-Aldrich, St. Louis, MO, USA). Pictures of the syncytia were taken using an OLYMPUS DP80 light microscope (Olympus, Tokyo, Japan), and the size of the syncytia was measured using Cellsens standard software.

### 4.3. Sequencing Sample Preparation

At 1 and 5 dpi, i.e., the early stages of soybean–SCN interactions for both resistant and susceptible cultivars, soybean roots were washed with running tape water and cleaned with sterilized water. Roots were then flash frozen in liquid nitrogen and stored in a −80 °C freezer. Three independent biological replicates were collected, and each replicate contained the roots of three soybean seedlings. The sampled roots were as follows: Non-inoculated W82 at 1 dpi (W1C), SCN-inoculated W82 at 1 dpi (W1N), non-inoculated HPZ at 1 dpi (H1C), and SCN-inoculated HPZ at 1 dpi (H1N). Likewise, the samples of W5C, W5N, H5C, and, H5N were taken at 5 dpi, giving a total of 24 samples.

### 4.4. RNA Isolation, Construction of Small RNA Libraries, and Sequencing

MicroRNA was extracted from all 24 samples with an EASYspin plant microRNA extraction kit (Aidlab biotechnologies Co., Ltd, Beijing, China), according to the manufacturer’s instructions. The purity, concentration, and integrity of the microRNA samples were tested using an Agilent Bioanalyzer 2100 (Agilent, Waldbronn, Germany). MicroRNA libraries were constructed using the NEB Next Multiplex Small RNA Library Prep Kit for Illumina. Then, these microRNA libraries were sequenced on an Illumina novaseq6000 at Biomarker company (Beijing, China), and 50 single-end reads were generated. All the raw data were uploaded to SRA (https://dataview.ncbi.nlm.nih.gov/object/PRJNA579002?reviewer=c9ctcl8rfej71udnds5o2jcn0g).

### 4.5. Analysis of Sequencing Data, Differential miRNA Expression Profiling, and Target Prediction

For data quality control, raw data (raw reads) in a fastq format were firstly processed through FastQC. In this step, clean reads were obtained by removing reads containing adapters, reads containing ploy-N, and low-quality reads. Further, reads were also trimmed and cleaned by removing the sequences smaller than 18 nt or longer than 30 nt. At the same time, Q30, GC-content, and sequence duplication levels of the clean data were calculated. All downstream analyses were based on high quality clean data. To comparatively analyze the 18 to 30 nt unannotated clean reads, Bowtie [65] was used to filter the clean reads using Silva database, GtRNAdb database, Rfam database, and Repbase database sequence alignment, and to filter out ribosomal RNAs (rRNA), transfer RNAs (tRNA), small nuclear RNAs (snRNA), small nucleolar RNAs (snoRNA), and other ncRNAs and repeats. The remaining reads were used to detect known miRNAs and novel miRNAs, which were predicted through comparisons with the soybean reference genome (*Glycine max*.Wm82.a2.v1) [32] and with known miRNAs from miRbase (v22) [33]. miRDeep2 [66] was used for novel miRNA prediction. The expression levels of the miRNAs in each sample were calculated and normalized using the TPM (transcripts per million) algorithm [67], and the raw counts of miRNAs were used for the analysis of differential miRNA expression that was conducted by using DESeq2(1.10.1) [68]. miRNAs that were found to have |log2(FC)| ≥ 1.00; FDR (False Discovery Rate) ≤ 0.01 by DESeq2 were assigned as differentially expressed. The potential target genes of the miRNAs were predicted using TargetFinder (v1.6), and these predicted target genes were then annotated by Blast to the NR, Swiss-Prot, GO, KEGG, and Pfam databases. KOG, GO, and KEGG pathway classification was implemented on the BMK cloud analysis platform.

### 4.6. Quantitative Real-Time PCR (qRT-PCR) Analysis to Validate miRNA Expression

To validate the expressions of the miRNAs identified in the sequencing analysis, three miRNAs with different expression patterns in different treatments were randomly selected for qRT-PCR assays. miRNA First Strand cDNA Synthesis (Sangon Biotech, Shanghai, China) was used for polyadenylation and reverse transcription of all miRNAS, according to the manufacturer’s instructions. The MicroRNAs qPCR Kit (Sangon Biotech, Shanghai) was used for miRNA qPCR with the following steps: 95 °C for 30 s, 40 cycles at 95 °C for 5 s, and 60 °C for 30 s. All qRT-PCR experiments were performed on the CFX Connect Real-Time PCR Detection System (Bio-Rad, Hercules, CA, USA). Soybean U6 snRNA was used as the internal reference gene for the miRNAs, and the data were quantified using the 2^−ΔΔCt^ method, all the primers used for qRT-PCR were shown in Supplementary Material S5. [69].

### 4.7. Statistical Analysis

Nematode infection, development, and syncytium size data were checked for normality and homogeneity of variance using the Kolmogorov–Smirnov or Shapiro–Wilk test. T-tests were then used to analyze the above data using SPSS software (SPSS Inc., Chicago, IL, USA).

## 5. Conclusions

SCN is the most damaging pathogen of soybean in terms of yield losses, and control strategies mainly rely on the few resistant cultivars available. The development of new technologies to study biological questions, such as high-throughput sequencing, CRISPR/Cas9 gene editing, and other novel technologies, will provide us with new methods to understanding the mechanisms behind how soybean responds to SCN infection, which we can use to manage SCN. In this study, we constructed 24 small RNA libraries for high-throughput sequencing, in order to investigate the response of soybean miRNAs to SCN in both a susceptible cultivar (W82) and a resistant cultivar (HPZ) at the early stages of infection (1 and 5 dpi). In total, 634 known miRNAs were identified, and 252 novel miRNAs were predicted. Among these miRNAs, a total of 14 known miRNAs belonging to 13 families and 26 novel miRNAs may be involved in the response to SCN infection, as they were differentially expressed in these 24 libraries constructed from HPZ and W82. These miRNAs included legume-specific miRNAs, conserved miRNAs, and novel predicted miRNAs (for instance, gma-miR3522, gma-miR408a, and novel_miR_106). Further analysis of the potential target genes of these DE miRNAs found that the target genes were highly related to plant defense, and that signal transduction, immune system processes, and nutrient metabolism were responsive to SCN invasion in KOG, GO, and KEGG pathway analysis. In summary, our findings provide new knowledge of the mechanisms behind soybean–SCN interaction at the miRNA level, and may act as a stepping stone for controlling the damage caused by SCN.

## Figures and Tables

**Table 1:** The raw reads and clean reads obtained in 24 small RNA libraries.

Sequence ID	Raw Reads	Clean Reads	Q30 (%)
W1C_1	27,715,234	20,070,645	96.82
W1C_2	27,484,434	21,474,884	96.78
W1C_3	26,580,279	20,491,433	94.98
W1N_1	30,925,791	19,880,712	96.79
W1N_2	31,646,226	22,819,271	95.36
W1N_3	42,045,429	23,714,940	97.49
H1C_1	48,930,291	33,148,086	94.54
H1C_2	43,635,613	19,622,055	94.57
H1C_3	58,984,552	31,856,657	94.94
H1N_1	55,931,293	36,612,046	96.78
H1N_2	44,159,489	28,178,164	96.89
H1N_3	44,233,050	27,250,451	96.87
W5C_1	47,456,852	32,505,320	96.94
W5C_2	46,321,364	21,402,542	96.90
W5C_3	37,909,448	23,830,730	96.84
W5N_1	39,218,867	20,669,157	94.60
W5N_2	51,829,668	38,402,767	94.18
W5N_3	51,434,403	34,331,810	94.77
H5C_1	43,331,598	32,478,323	94.71
H5C_2	46,365,046	35,279,570	94.27
H5C_3	50,242,257	32,787,129	94.90
H5N_1	33,241,813	25,374,292	96.39
H5N_2	42,384,481	20,954,527	96.90
H5N_3	48,968,205	20,332,827	96.97
Total reads	1,020,975,683	643,468,338	

**Table 2:** Known miRNA identified and novel miRNA predicted in 24 small RNA libraries.

Sequence ID	Known-miRNAs	Novel-miRNAs	Total
W1C_1	481	230	711
W1C_2	489	232	721
W1C_3	521	242	763
W1N_1	471	214	685
W1N_2	475	227	702
W1N_3	494	213	707
H1C_1	547	243	790
H1C_2	514	241	755
H1C_3	552	242	794
H1N_1	540	245	785
H1N_2	508	238	746
H1N_3	512	235	747
W5C_1	552	240	792
W5C_2	495	238	733
W5C_3	525	249	774
W5N_1	502	239	741
W5N_2	541	249	790
W5N_3	555	249	804
H5C_1	536	244	780
H5C_2	529	247	776
H5C_3	534	248	782
H5N_1	519	232	751
H5N_2	517	240	757
H5N_3	507	244	751
Total	634	252	886

The identification of known miRNAs by comparing with soybean reference genome (*Glycine max*.Wm82.a2.v1) and known miRNAs from miRbase (v22); miRDeep2 was used for novel miRNA prediction.

**Table 3:** The number of differentially expressed miRNAs in each comparison.

DE Set	Total DE miRNA	Upregulated	Downregulated
W1C_1_W1C_2_W1C_3 vs W1N_1_W1N_2_W1N_3	11	9	2
H1C_1_H1C_2_H1C_3 vs H1N_1_H1N_2_H1N_3	7	2	5
W5C_1_W5C_2_W5C_3 vs W5N_1_W5N_2_W5N_3	3	3	0
H5C_1_H5C_2_H5C_3 vs H5N_1_H5N_2_H5N_3	3	3	0
W1N_1_W1N_2_W1N_3 vs W5N_1_W5N_2_W5N_3	11	3	8
H1N_1_H1N_2_H1N_3 vs H5N_1_H5N_2_H5N_3	9	6	3
W1N_1_W1N_2_W1N_3 vs H1N_1_H1N_2_H1N_3	10	3	7
W5N_1_W5N_2_W5N_3 vs H5N_1_H5N_2_H5N_3	9	0	9

miRNA differential expression analysis was conducted by DESeq2 (1.10.1), miRNA with |log2(FC)| ≥ 1.00; FDR (False Discovery Rate) ≤ 0.01 found by DESeq2 were assigned as differentially expressed.

**Table 4:** Function annotation of the potential target genes of differentially expressed miRNAs.

DE Set	miRNA	Target Gene	Function Annotation
W1C vs W1N	gma-miR408a-5p	Glyma.04G248700.Wm82.a2.v1	Xylanase inhibitor N-terminal
	gma-miR408a-5p	Glyma.06G114200.Wm82.a2.v1	Xylanase inhibitor N-terminal
	gma-miR408a-5p	Glyma.07G103400.Wm82.a2.v1	Protein tyrosine kinase
	gma-miR408a-5p	Glyma.09G174000.Wm82.a2.v1	Protein tyrosine kinase
	gma-miR408a-5p	Glyma.10G282000.Wm82.a2.v1	Ubiquitin-conjugating enzyme
	gma-miR408a-5p	Glyma.20G107300.Wm82.a2.v1	Ubiquitin-conjugating enzyme
	gma-miR4415a-3p	Glyma.13G076900.Wm82.a2.v1	Multicopper oxidase
	gma-miR4415a-3p	Glyma.14G041300.Wm82.a2.v1	Multicopper oxidase
	gma-miR4415a-3p	Glyma.20G051700.Wm82.a2.v1	Multicopper oxidase
	gma-miR4415a-3p	Glyma.20G051900.Wm82.a2.v1	Multicopper oxidase
	gma-miR4415a-3p	Glyma.20G051600.Wm82.a2.v1	Multicopper oxidase
	gma-miR4415a-3p	Glyma.20G051700.Wm82.a2.v1	Multicopper oxidase
	gma-miR4415a-3p	Glyma.20G051900.Wm82.a2.v1	Multicopper oxidase
H1C vs H1N	novel_miR_63	Glyma.05G145000.Wm82.a2.v1	ABC transporter
	novel_miR_63	Glyma.08G101500.Wm82.a2.v1	ABC transporter
	novel_miR_63	Glyma.08G149300.Wm82.a2.v1	Glycosyl hydrolases family 28
	novel_miR_63	Glyma.10G231100.Wm82.a2.v1	Glycosyl hydrolases family 28
	novel_miR_63	Glyma.15G269400.Wm82.a2.v1	Glycosyl hydrolases family 28
	novel_miR_237	Glyma.05G145000.Wm82.a2.v1	ABC transporter
	novel_miR_237	Glyma.08G101500.Wm82.a2.v1	ABC transporter
	novel_miR_237	Glyma.08G149300.Wm82.a2.v1	Glycosyl hydrolases family 28
	novel_miR_237	Glyma.10G231100.Wm82.a2.v1	Glycosyl hydrolases family 28
	novel_miR_237	Glyma.15G269400.Wm82.a2.v1	Glycosyl hydrolases family 28
	gma-miR3522	Glyma.04G121700.Wm82.a2.v1	Polyphenol oxidase middle domain
	gma-miR3522	Glyma.07G193300.Wm82.a2.v1	Polyphenol oxidase middle domain
	gma-miR3522	Glyma.07G193500.Wm82.a2.v1	Polyphenol oxidase middle domain
	gma-miR3522	Glyma.13G183200.Wm82.a2.v1	Polyphenol oxidase middle domain
	gma-miR3522	Glyma.15G071200.Wm82.a2.v1	Polyphenol oxidase middle domain
	gma-miR3522	Glyma.05G167100.Wm82.a2.v1	Neprosin activation peptide
	gma-miR3522	Glyma.08G125400.Wm82.a2.v1	Neprosin activation peptide
W5C vs W5N	gma-miR408a-3p	Glyma.02G231600.Wm82.a2.v1	Multicopper oxidase
	gma-miR408a-3p	Glyma.02G261600.Wm82.a2.v1	Multicopper oxidase
	gma-miR408a-3p	Glyma.11G164000.Wm82.a2.v1	Multicopper oxidase
	gma-miR408a-3p	Glyma.11G233400.Wm82.a2.v1	Multicopper oxidase
	gma-miR408a-3p	Glyma.14G056100.Wm82.a2.v1	Multicopper oxidase
	gma-miR408a-3p	Glyma.14G198900.Wm82.a2.v1	Multicopper oxidase
	gma-miR408a-3p	Glyma.18G023600.Wm82.a2.v1	Multicopper oxidase
	gma-miR408a-3p	Glyma.18G057200.Wm82.a2.v1	Multicopper oxidase
	gma-miR408a-3p	Glyma.03G189800.Wm82.a2.v1	Leucine Rich repeats
	gma-miR408a-3p	Glyma.06G142500.Wm82.a2.v1	Leucine Rich repeats
	gma-miR408a-3p	Glyma.19G190200.Wm82.a2.v1	Leucine rich repeat
	novel_miR_178	Glyma.04G103900.Wm82.a2.v1	AP2 domain
	novel_miR_178	Glyma.20G224000.Wm82.a2.v1	Myb-like DNA-binding domain
H5C vs H5N	novel_miR_106	Glyma.01G031500.Wm82.a2.v1	Aldehyde dehydrogenase family
	novel_miR_106	Glyma.02G034000.Wm82.a2.v1	Aldehyde dehydrogenase family
	novel_miR_106	Glyma.16G168700.Wm82.a2.v1	Leucine rich repeat
	novel_miR_106	Glyma.15G245900.Wm82.a2.v1	Leucine rich repeat
	novel_miR_106	Glyma.17G250800.Wm82.a2.v1	Leucine rich repeat
	novel_miR_106	Glyma.09G184300.Wm82.a2.v1	Serine hydroxymethyltransferase
	novel_miR_34	Glyma.01G031800.Wm82.a2.v1	K+ potassium transporter
	novel_miR_34	Glyma.02G033600.Wm82.a2.v1	K+ potassium transporter
	novel_miR_34	Glyma.04G200600.Wm82.a2.v1	Auxin response factor
	novel_miR_34	Glyma.06G164900.Wm82.a2.v1	Auxin response factor
W1N vs W5N	gma-miR159a-5p	Glyma.01G183300.Wm82.a2.v1	NB-ARC domain
	gma-miR159a-5p	Glyma.15G230900.Wm82.a2.v1	NB-ARC domain
	gma-miR159a-5p	Glyma.06G134200.Wm82.a2.v1	Protein kinase domain
	gma-miR159a-5p	Glyma.06G258300.Wm82.a2.v1	Protein kinase domain
	gma-miR5037c	Glyma.01G005400.Wm82.a2.v1	Phosphofructokinase
	gma-miR5037c	Glyma.04G139400.Wm82.a2.v1	Plant calmodulin-binding domain
	gma-miR5037c	Glyma.20G154800.Wm82.a2.v1	GMC oxidoreductase
	gma-miR5037c	Glyma.U040400.Wm82.a2.v1	GMC oxidoreductase
	gma-miR5037c	Glyma.08G275900.Wm82.a2.v1	mTERF
	gma-miR5037c	Glyma.08G306000.Wm82.a2.v1	mTERF
	gma-miR5225	Glyma.15G252700.Wm82.a2.v1	Protein tyrosine kinase
	gma-miR5225	Glyma.19G130400.Wm82.a2.v1	VQ motif
	novel_miR_13	Glyma.05G232000.Wm82.a2.v1	Leucine rich repeat
	novel_miR_13	Glyma.08G039400.Wm82.a2.v1	Leucine rich repeat
	novel_miR_13	Glyma.07G103400.Wm82.a2.v1	Protein tyrosine kinase
W1N vs H1N	novel_miR_120	Glyma.01G181900.Wm82.a2.v1	Cytochrome P450
	novel_miR_120	Glyma.09G279100.Wm82.a2.v1	Cytochrome P450
	novel_miR_142	Glyma.04G135400.Wm82.a2.v1	Myb-like DNA-binding domain
	novel_miR_142	Glyma.18G159300.Wm82.a2.v1	Leucine rich repeat
	novel_miR_200	Glyma.20G046100.Wm82.a2.v1	NB-ARC domain
	novel_miR_200	Glyma.20G046200.Wm82.a2.v1	NB-ARC domain
	novel_miR_200	Glyma.01G112600.Wm82.a2.v1	Multicopper oxidase
	novel_miR_200	Glyma.07G133900.Wm82.a2.v1	Multicopper oxidase
W5N vs H5N	gma-miR398d	Glyma.05G055000.Wm82.a2.v1	Copper/zinc superoxide dismutase (SODC)
	gma-miR398d	Glyma.11G236800.Wm82.a2.v1	Multicopper oxidase
	gma-miR862b	Glyma.01G062400.Wm82.a2.v1	Copper amine oxidase
	gma-miR862b	Glyma.01G063700.Wm82.a2.v1	SBP domain
	gma-miR862b	Glyma.02G121300.Wm82.a2.v1	SBP domain
	gma-miR862b	Glyma.04G254300.Wm82.a2.v1	Serine hydroxymethyltransferase
	gma-miR862b	Glyma.06G107800.Wm82.a2.v1	Serine hydroxymethyltransferase
	gma-miR862b	Glyma.10G036700.Wm82.a2.v1	AP2 domain
	gma-miR862b	Glyma.13G123100.Wm82.a2.v1	AP2 domain
	gma-miR862b	Glyma.11G166300.Wm82.a2.v1	Glutaredoxin
	gma-miR862b	Glyma.11G232300.Wm82.a2.v1	Glutaredoxin
	novel_miR_105	Glyma.01G043300.Wm82.a2.v1	WRKY DNA -binding domain
	novel_miR_105	Glyma.13G365600.Wm82.a2.v1	Glycosyl hydrolases family 17
	novel_miR_105	Glyma.15G007600.Wm82.a2.v1	Glycosyl hydrolases family 17

The potential target genes of the miRNAs were predicted using TargetFinder (v1.6), and these predicted target genes were then annotated by Blast to the NR, Swiss-Prot, GO, KEGG, and Pfam databases.

## Supplementary Materials

Supplementary materials can be found at https://www.mdpi.com/1422-0067/20/22/5634/s1. Supplementary Material S1 sRNA classification. Supplementary Material S2 TPM value of miRNAs. Supplementary Material S3 all DE miRNAs. Supplementary Material S4 all DEmiRNAs and potential target genes. Supplementary Material S5 primers used for qRT-PCR.

## Abbreviations

SCN	soybean cyst nematode
miRNA	microRNA
qRT-PCR	quantitative real-time PCR

## References

[1] Whitham S.A., Qi M., Innes R.W., Ma W., Lopescaitar V.S., Hewezi T. (2016). Molecular Soybean-Pathogen Interactions. Annu. Rev. Phytopathol..

[2] Wang H., Zhao H., Chu D. (2015). Genetic structure analysis of populations of the soybean cyst nematode, *Heterodera glycines*, from north China. Nematology.

[3] Koenning S.R., Wrather J.A. (2010). Suppression of Soybean Yield Potential in the Continental United States by Plant Diseases from 2006 to 2009. Plant Health Progress..

[4] Concibido V.C., Diers B.W., Arelli P.R. (2004). A decade of QTL mapping for cyst nematode resistance in soybean. Crop Sci..

[5] Mitchum M.G. (2016). Soybean resistance to the soybean cyst nematode *Heterodera glycines*: An update. Phytopathology.

[6] Cook D.E., Lee T.G., Guo X., Melito S., Wang K., Bayless A.M., Wang J., Hughes T.J., Willis D.K., Clemente T.E. (2012). Copy Number Variation of Multiple Genes at *Rhg1* Mediates Nematode Resistance in Soybean. Science.

[7] Carthew R.W., Sontheimer E.J. (2009). Origins and Mechanisms of miRNAs and siRNAs. Cell.

[8] Chen X. (2009). Small RNAs and Their Roles in Plant Development. Annu. Rev. Cell Dev. Biol..

[9] Bologna N.G., Voinnet O. (2014). The Diversity, Biogenesis, and Activities of Endogenous Silencing Small RNAs in *Arabidopsis*. Annu. Rev. Plant Biol..

[10] Chitwood D.H., Sinha N.R. (2014). Plant Development: Small RNAs and the Metamorphosis of Leaves. Curr. Biol..

[11] Balyan S.C., Mutum R.D., Kansal S., Kumar S., Raghuvanshi S. (2015). Insights into the small RNA-mediated networks in response to abiotic stress in plants. Elucidation of Abiotic Stress Signaling in Plants.

[12] Curaba J., Singh M.B., Bhalla P.L. (2014). miRNAs in the crosstalk between phytohormone signalling pathways. J. Exp. Bot..

[13] Zhang H.L., Chen L., Li W.N., Wang L.L., Xie H.Y. (2014). Plant MicroRNAs Responsive to Fungal Infection. Adv. Mater. Res..

[14] Allen E., Xie Z., Gustafson A.M., Carrington J.C. (2005). microRNA-Directed Phasing during Trans-Acting siRNA Biogenesis in Plants. Cell.

[15] Achkar N.P., Cambiagno D.A., Manavella P.A. (2016). miRNA Biogenesis: A Dynamic Pathway. Trends Plant Sci..

[16] Park M.Y., Wu G., Gonzalezsulser A., Vaucheret H., Poethig R.S. (2005). Nuclear processing and export of microRNAs in Arabidopsis. Proc. Natl. Acad. Sci. USA.

[17] German M.A., Pillay M., Jeong D., Hetawal A., Luo S., Janardhanan P.E., Kannan V., Rymarquis L.A., Nobuta K., German R. (2008). Global identification of microRNA-target RNA pairs by parallel analysis of RNA ends. Nat. Biotechnol..

[18] Navarro L., Dunoyer P., Jay F., Arnold B.C., Dharmasiri N., Estelle M., Voinnet O., Jones J.D.G. (2006). A plant miRNA contributes to antibacterial resistance by repressing auxin signaling. Science.

[19] Fahlgren N., Howell M.D., Kasschau K.D., Chapman E.J., Sullivan C.M., Cumbie J.S., Givan S.A., Law T.F., Grant S.R., Dangl J.L. (2007). High-throughput sequencing of Arabidopsis microRNAs: Evidence for frequent birth and death of *MIRNA* genes. PLoS ONE.

[20] He X., Fang Y., Feng L., Guo H. (2008). Characterization of conserved and novel microRNAs and their targets, including a TuMV-induced TIR–NBS–LRR class R gene-derived novel miRNA in *Brassica*. FEBS Lett..

[21] Zhang T., Zhao Y., Zhao J., Wang S., Jin Y., Chen Z., Fang Y., Hua C., Ding S., Guo H. (2016). Cotton plants export microRNAs to inhibit virulence gene expression in a fungal pathogen. Nat. Plants.

[22] Medina C., Rocha M.D., Magliano M., Ratpopoulo A., Revel B., Marteu N., Magnone V., Lebrigand K., Cabrera J., Barcala M. (2017). Characterization of microRNAs from *Arabidopsis* galls highlights a role for miR159 in the plant response to the root-knot nematode *Meloidogyne incognita*. New Phytol..

[23] Combier J., Frugier F., De Billy F., Boualem A., Elyahyaoui F., Moreau S., Vernie T., Ott T., Gamas P., Crespi M. (2006). MtHAP2-1 is a key transcriptional regulator of symbiotic nodule development regulated by microRNA169 in *Medicago truncatula*. Genes Dev..

[24] Li X., Xue W., Shaopeng Z., Dawei L., Yuxi D., Wei D., Baohong Z. (2012). Identification of Soybean MicroRNAs Involved in Soybean Cyst Nematode Infection by Deep Sequencing. PLoS ONE.

[25] Xu M., Li Y., Zhang Q., Xu T., Qiu L., Fan Y., Wang L. (2014). Novel miRNA and phasiRNA biogenesis networks in soybean roots from two sister lines that are resistant and susceptible to SCN race 4. PLoS ONE.

[26] Tian B., Shichen W., Todd T.C., Johnson C.D., Tang G., Trick H.N. (2017). Genome-wide identification of soybean microRNA responsive to soybean cyst nematodes infection by deep sequencing. BMC Genomics.

[27] Wong J., Gao L., Yang Y., Zhai J., Arikit S., Yu Y., Duan S., Chan V., Xiong Q., Yan J. (2014). Roles of small RNAs in soybean defense against *Phytophthora sojae* infection. Plant J..

[28] Li S., Chen Y., Zhu X., Wang Y., Jung K.-H., Chen L., Xuan Y., Duan Y. (2018). The transcriptomic changes of Huipizhi Heidou(*Glycine max*), a nematode-resistant black soybean during *Heterodera glycines* race 3 infection. J. Plant Physiol..

[29] Kim Y.H., Riggs R.D., Kim K.S. (1987). Structural Changes Associated with Resistance of Soybean to *Heterodera glycines*. J. Nematol..

[30] Handoo Z.A., Anand S.C. (1993). Biological manifestation of resistance to soybean cyst nematode development in ‘Hartwig’ soybean. Crop Prot..

[31] Riggs R.D. (1973). Ultrastructural Changes in Peking Soybeans Infected With *Heterodera glycines*. Phytopathology.

[32] Schmutz J., Cannon S.B., Schlueter J., Ma J., Mitros T., Nelson W., Hyten D.L., Song Q., Thelen J.J., Cheng J. (2010). Genome sequence of the palaeopolyploid soybean. Nature.

[33] Griffithsjones S., Grocock R.J., Dongen S.V., Bateman A., Enright A.J. (2006). miRBase: microRNA sequences, targets and gene nomenclature. Nucleic Acids Res..

[34] Chen C., Xia R., Chen H., He Y. (2018). TBtools, a Toolkit for Biologists integrating various HTS-data handling tools with a user-friendly interface. bioRxiv.

[35] Jiang N., Meng J., Cui J., Sun G., Luan Y. (2018). Function identification of miR482b, a negative regulator during tomato resistance to *Phytophthora infestans*. Hortic. Res..

[36] Dai Z., Tan J., Zhou C., Yang X., Yang F., Zhang S., Sun S., Miao X., Shi Z. (2019). The OsmiR396-*OsGRF8*-*OsF3H*-flavonoid pathway mediates resistance to the brown planthopper in rice (*Oryza sativa*). Plant Biotechnol. J..

[37] Ding Y., Ma Y., Liu N., Xu J., Hu Q., Li Y., Wu Y., Xie S., Zhu L., Min L. (2017). microRNAs involved in auxin signalling modulate male sterility under high-temperature stress in cotton (*Gossypium hirsutum*). Plant J..

[38] Hewezi T., Howe P., Maier T., Baum T.J. (2008). *Arabidopsis* small RNAs and their targets during cyst nematode parasitism. Mol. Plant-Microbe Interact..

[39] Cabrera J., Barcala M., Garcia A., Riomachin A., Medina C., Jaubertpossamai S., Favery B., Maizel A., Ruizferrer V., Fenoll C. (2016). Differentially expressed small RNAs in Arabidopsis galls formed by *Meloidogyne javanica*: A functional role for miR390 and its *TAS3*-derived tasiRNAs. New Phytol..

[40] Koter M.D., Świecicka M., Matuszkiewicz M., Pacak A., Derebecka N., Filipecki M. (2018). The miRNAome dynamics during developmental and metabolic reprogramming of tomato root infected with potato cyst nematode. Plant Sci..

[41] Kaur P., Shukla N., Joshi G., Vijayakumar C., Kumar A. (2017). Genome-wide identification and characterization of miRNAome from tomato (*Solanum lycopersicum*) roots and root-knot nematode (*Meloidogyne incognita*) during susceptible interaction. PLoS ONE.

[42] Huang J., Meiling Y., Lu L., Xiaoming Z. (2016). Diverse Functions of Small RNAs in Different Plant–Pathogen Communications. Front. Microbiol..

[43] Li L., Steffens J.C. (2002). Overexpression of polyphenol oxidase in transgenic tomato plants results in enhanced bacterial disease resistance. Planta.

[44] Masato K., Akiko O.O., Yutaka A., Takanobu Y., Koji A., Tohru T., Tsutomu A. (2011). GMC oxidoreductase, a highly expressed protein in a potent biocontrol agent *Fusarium oxysporum* Cong:1-2, is dispensable for biocontrol activity. J. Gen. Appl. Microbiol..

[45] Hsiao Y.M., Liu Y.F., Lee P.Y., Hsu P.C., Pan Y.C. (2011). Functional Characterization of *copA* Gene Encoding Multicopper Oxidase in *Xanthomonas campestris* pv. campestris. J. Appl. Microbiol..

[46] Zhang W., Gao S., Zhou X., Chellappan P., Chen Z., Zhou X., Zhang X., Fromuth N., Coutino G., Coffey M. (2011). Bacteria-responsive microRNAs regulate plant innate immunity by modulating plant hormone networks. Plant Mol. Biol..

[47] Chen W., Sudisha J., WenYing Z., Mostafa A., Gui F.J. (2018). Spatio-temporal expression of miRNA159 family members and their *GAMYB* target gene during the modulation of gibberellin-induced grapevine parthenocarpy. J. Exp. Bot..

[48] Guan Q., Lu X., Zeng H., Zhang Y., Zhu J. (2013). Heat stress induction of miR398 triggers a regulatory loop that is critical for thermotolerance in Arabidopsis. Plant J..

[49] Xu W., Meng Y., Wise R.P. (2014). Mla- and Rom1- mediated control of microRNA398 and chloroplast copper/zinc superoxide dismutase regulates cell death in response to the barley powdery mildew fungus. New Phytol..

[50] Sunkar R., Kapoor A., Zhu J. (2006). Posttranscriptional Induction of Two Cu/Zn Superoxide Dismutase Genes in *Arabidopsis* Is Mediated by Downregulation of miR398 and Important for Oxidative Stress Tolerance. Plant Cell..

[51] Guo Z.J., Chen X.J., Wu X.L., Ling J.Q., Xu P. (2004). Overexpression of the AP2/EREBP transcription factor *OPBP1* enhances disease resistance and salt tolerance in tobacco. Plant Mol. Biol..

[52] Jones D.A., Jones J.D.G. (1997). The Role of Leucine-Rich Repeat Proteins in Plant Defences. Adv. Bot. Res..

[53] Reinprecht Y., Yadegari Z., Perry G.E., Siddiqua M., Wright L.C., McClean P.E., Pauls K.P. (2013). In silico comparison of genomic regions containing genes coding for enzymes and transcription factors for the phenylpropanoid pathway in *Phaseolus vulgaris* L. and *Glycine max* L.. Front. Plant Sci..

[54] Siddique S., Grundler F.M.W. (2015). Metabolism in Nematode Feeding Sites. Adv. Bot. Res..

[55] Díaz-Manzano F.E., Cabrera J., Ripoll J.J., del Olmo I., Andrés M.F., Silva A.C., Barcala M., Sánchez M., Ruíz-Ferrer V., de Almeida-Engler J. (2018). A role for the gene regulatory module microRNA172/TARGET OF EARLY ACTIVATION TAGGED 1/FLOWERING LOCUS T (miRNA 172/*TOE 1*/*FT*) in the feeding sites induced by *Meloidogyne javanica* in *Arabidopsis thaliana*. New Phytol..

[56] Zhao W., Li Z., Fan J., Hu C., Yang R., Qi X., Chen H., Zhao F., Wang S. (2015). Identification of jasmonic acid-associated microRNAs and characterization of the regulatory roles of the miR319/*TCP4* module under root-knot nematode stress in tomato. J. Exp. Bot..

[57] Jaubertpossamai S., Noureddine Y., Favery B. (2019). MicroRNAs, new players in the plant-nematode interaction. Front. Plant Sci..

[58] Wang M., Weiberg A., Dellota E., Yamane D., Jin H. (2017). Botrytis small RNA Bc-siR37 suppresses plant defense genes by cross-kingdom RNAi. RNA Biol..

[59] Yan J., Gu Y., Jia X., Kang W., Pan S., Tang X., Chen X., Tang G. (2012). Effective Small RNA Destruction by the Expression of a Short Tandem Target Mimic in *Arabidopsis*. Plant Cell..

[60] Nizampatnam N.R., Schreier S.J., Damodaran S., Adhikari S. (2015). microRNA160 dictates stage-specific auxin and cytokinin sensitivities and directs soybean nodule development. Plant J..

[61] Sun X., Hu Z., Chen R., Jiang Q., Song G., Zhang H., Xi Y. (2015). Targeted mutagenesis in soybean using the CRISPR-Cas9 system. Sci. Rep..

[62] Mahalingam R., Knap H.T., Lewis S.A. (1998). Inoculation Method for Studying Early Responses of *Glycine max* to *Heterodera glycines*. J. Nematol..

[63] Faghihi J., Ferris J.M. (2000). An efficient new device to release eggs from *Heterodera glycines*. J. Nematol..

[64] Bybd D.W., Kirkpatrick T.E., Barker K.R. (1983). An Improved Technique for Clearing and Staining Plant Tissues for Detection of Nematodes. J. Nematol..

[65] Langmead B., Trapnell C., Pop M., Salzberg S.L. (2009). Ultrafast and memory-efficient alignment of short DNA sequences to the human genome. Genome Biol..

[66] Friedlander M.R., Mackowiak S.D., Li N., Chen W.Y., Rajewsky N. (2012). miRDeep2 accurately identifies known and hundreds of novel microRNA genes in seven animal clades. Nucleic Acids Res..

[67] Li B., Ruotti V., Stewart R., Thomson J.A., Dewey C.N. (2010). RNA-Seq gene expression estimation with read mapping uncertainty. Bioinformatics.

[68] Love M.I., Huber W., Anders S. (2014). Moderated estimation of fold change and dispersion for RNA-seq data with DESeq2. Genome Biol..

[69] Livak K.J., Schmittgen T.D. (2001). Analysis of relative gene expression data using real-time quantitative PCR and the 2(-Delta Delta C(T)) Method. Methods.

